# The Inhibitory Effect of WenxinKeli on H9C2 Cardiomyocytes Hypertrophy Induced by Angiotensin II through Regulating Autophagy Activity

**DOI:** 10.1155/2017/7042872

**Published:** 2017-06-20

**Authors:** Jie Li, Yang Li, Ying Zhang, Dan Hu, Yonghong Gao, Hongcai Shang, Yanwei Xing

**Affiliations:** ^1^Guang'anmen Hospital, Chinese Academy of Chinese Medical Sciences, Beijing 100053, China; ^2^Key Laboratory of Chinese Internal Medicine of the Ministry of Education, Dongzhimen Hospital Affiliated to Beijing University of Chinese Medicine, Beijing 100700, China; ^3^Department of Cardiology, General Hospital of People's Liberation Army, Beijing 100853, China; ^4^Xuan Wu TCM Hospital, Beijing 100053, China; ^5^Masonic Medical Research Laboratory, Utica, NY 13501, USA

## Abstract

**Objectives:**

We investigated the role of cardiomyocyte autophagy and its regulatory mechanisms by WenxinKeli (WXKL) in cells subjected to hypertrophy.

**Methods:**

H9C2 cardiomyocytes were divided into 8 groups. Cytoskeletal proteins as well as endogenously expressed autophagy marker proteins were studied by confocal imaging. Western blotting was used to assess the levels of light chain-3 (LC3) and mechanistic target of rapamycin (mTOR). The cell viability assay was used to detect the content of ATP. Flow cytometry was used to detect apoptotic cardiomyocytes.

**Results:**

(1) Compared with the control group, the length and width of cells in the Angiotensin II (AngII) group were significantly increased, while those in the 3-methyladenine (3-MA) and the WXKL groups were decreased. (2) Compared with AngII group, the expression of LC3 II/I protein in the 3-MA and WXKL groups was downregulated, while the expression of mTOR protein was upregulated. (3) Compared with the AngII group, the cardiomyocytes in the WXKL group showed increased ATP and decreased apoptosis rate and number of autophagosome.

**Conclusions:**

We propose a novel role of WXKL as a likely inhibitor of cardiac hypertrophy by regulation of pathological autophagy.

## 1. Introduction

Epidemiological data show that heart failure is a leading cause of morbidity and mortality worldwide [1]. Although drugs and other therapies have been developed for the management of heart failure, its 5-year mortality rate remains high [2, 3]. Previous studies have revealed that heart failure is associated with cardiac remodeling [4]. Cardiac remodeling is a chronic adaptation process, its characteristics include progressive ventricular expansion, myocardial hypertrophy, fibrosis, and cardiac performance deterioration. It is amended by the adaptability of cardiomyocytes, as well as the adaptability of the negative aspects, such as the interaction between myocardial cell death and fibrosis [5]. Hypertrophy and fibrosis obstruct cardiac microcirculation, resulting in tissue hypoxia and subsequent loss of cardiomyocytes. Consequently, cardiac function is continuously reduced and hypertrophy gradually transitions to heart failure, which is progressive and accompanied by a growing enlargement of the ventricular cavity with fibrosis and disarray. Accordingly, investigation of the mechanism of cardiac hypertrophy could help us to discover targets for the prevention and cure of heart failure.

Autophagy is a highly conserved process from yeast to mammals for the bulk degradation and recycling of proteins and lipids. Autophagy is critical for organelle biogenesis and turnover, cell growth, and development [6, 7]. Increasing evidence demonstrates that autophagy plays a crucial role in cardiac remodeling to maintain cardiac function and cellular homeostasis in the heart [8, 9]. One study showed that temporally controlled cardiac-specific deficiency of Atg5, a protein required for autophagy, led to cardiac hypertrophy [3]. Under stress stimulation, autophagy acts either as a protective factor for cardiomyocyte survival or as a risk factor for cardiomyocyte death. Basal autophagy occurs constitutively in the heart and is markedly upregulated to protect the heart during ischemic cardiomyopathy, heart failure, and cardiac hypertrophy [10]. However, excessive activation of autophagy can cause degradation of a large number of organelles and cell death [11, 12]. Thus, a level of autophagic activity above or below normal levels of the cardiac function and homeostasis is detrimental to cardiac function. However, the mechanism that regulates autophagy in cardiomyocyte remains unclear. Angiotensin II (AngII) plays an important role in the onset and development of cardiac remodeling. Research showed that AngII could lead to myocardial remodeling and upregulate myocardial autophagy. Then, whether AngII leads to myocardial remodeling through upregulation of myocardial autophagy or not remains to be answered.

WenxinKeli (WXKL) was prepared by Guang'anmen Hospital (Chinese Academy of Chinese Medical Sciences). The clinical experiment showed that WXKL has satisfactory curative effects in the treatment of chronic heart failure and arrhythmia [13]. WXKL contains five ingredients, namely, *Codonopsis, Polygonatum, Panax, nard*, and *amber*. A large number of studies [14] have demonstrated that WXKL can increase coronary blood flow, reduce myocardial oxygen consumption, enhance myocardial compliance, improve myocardial hypoxia tolerance, relieve anterior and posterior cardiac loading, reduce myocardial tissue damage in patients with high blood pressure, and reduce the occurrence of arrhythmia. Studies have also shown that WXKL produces atrial-selective depression of I_Na_-dependent parameters in canine isolated coronary-perfused preparations via a unique mechanism and is effective in both suppressing atrial fibrillation (AF) and preventing its induction [15]. The latest study indicated that WXKL significantly reduced the expression of CaMkinaseII (CaMKII), p-CaMKII (Thr-286), and phospholamban (Plb), but significantly increased the expression of the ryanodine receptor 2 (Ryr2), phosphorylated Plb (p-Plb), and FKBP12.6 in rats with myocardial infarction to improve cardiac function and inhibit myocardial remodeling [16]. However, to our knowledge, whether WXKL alleviates cardiac hypertrophy through the regulation of myocardial autophagy has not been investigated. Here, we selected rat embryonic cardiomyocyte cells and used protein detection, flow cytometry, laser confocal, and other methods to investigate the potential of WXKL to attenuate cardiac hypertrophy by inducing protective autophagy.

## 2. Materials and Methods

### 2.1. Cell Culture

The H9C2 rat embryonic cardiomyocyte cell line was derived from a rat embryonic heart subclone cell line and purchased from the Institute of Basic Medical Sciences, Chinese Academy of Medical Sciences (Cell Resource Center, IBMS, CAMS/PUMC).

### 2.2. Drugs and Solutions

WXKL was obtained from Shandong Buchang Pharmaceuticals Co., Ltd., China (Lot number: 120620). WXKL, consisting of *Rhizoma nardostachyos*, *Codonopsis*, *Notoginseng*, amber, and *Rhizoma Polygonati*, has been approved by the National Pharmacopoeia (National Pharmacopoeia Committee, 2005). According to the National Pharmacopoeia, the total amount of notoginseng saponin R1 (C_47_H_80_O_18_), ginseng saponin Rg1 (C_42_H_72_O_14_), and ginseng saponin Rb1 (C_54_H_92_O_23_) should not be less than 17 mg per bag (9 g) [16]. The drug was dissolved in saline solution at concentrations of 1 g/L, 5 g/L, and 10 g/L prior to the experiment. Rapamycin (Rapa, 37094; Sigma Co., Saint Louis, MO, USA) was made up in ethanol at 100 nM. The 3-methyladenine (3-MA) (M9281; Sigma Co.) was dissolved in deionized water at 10 mM. AngII (A9525, Sigma Co.) was dissolved in phosphate-buffered saline (PBS) at a concentration of 100 nmol/L.

### 2.3. Cell Grouping and Administration of Drugs

The H9C2 rat embryonic cardiomyocyte cells were divided into 8 different treatment groups, as follows: (1) control group: H9C2 cells were cultured in DMEM for 24 h. (2) EBSS group: H9C2 cells were cultured in EBSS for 3 h. (3) AngII group: H9C2 cells were cultured with 7–10 mol/L AngII for 24 h. (4) AngII + 1 g/L WXKL group: H9C2 cells were cultured with AngII for 24 h, and then 1 g/L of WXKL was added and cells were cultured for another 24 h. (5) AngII + 5 g/L WXKL group: H9C2 cells were cultured with AngII for 24 h, and then 5 g/L of WXKL was added and cells were cultured for another 24 h. (6) AngII + 10 g/L WXKL group: H9C2 cells were cultured with AngII for 24 h, and then 10 g/L of WXKL was added and cells were cultured for another 24 h. (7) AngII + 3-MA: H9C2 cells were pretreated with 3-MA for 3 h, and then AngII was added and cells were cultured for another 24 h. (8) Rapa group: H9C2 cells were cultured with Rapa for 24 h.

### 2.4. Western Blot Analysis

After treatment with the indicated condition, cells were harvested and washed with cold phosphate-buffered saline (PBS) and followed by incubation in Cell Lysis Buffer (Beyotime Biotechnology, China) in ice for at least 20 min. The lysates were centrifuged at 12,000 ×g for 10 min, and the supernatants were collected. Equal amount of protein was run on SDS-PAGE gels and subsequently electro-transferred to polyvinylidene fluoride (PVDF) membranes. The membrane was washed 3 times with TBST. Then, the primary antibodies were diluted with the Western antibody dilutions and incubated at 4°C in a horizontal shaker overnight, dilution ratio: Anti-MAP1LC3A antibody (1 : 2500; Abcam, Cambridge, England, UK) and Anti-mTOR antibody (1 : 1000; Abcam). The secondary antibody was diluted with 5% skim milk powder and incubated at room temperature for 60 min, ImmunoPure goat anti-rabbit IgG antibody (H+L) (1 : 5000; Thermo-Fisher, Waltham, MA, USA). Then we lifted the PVDF membrane and drained the excess detergent solution. The film was wrapped with a clean plastic wrap and placed in the development folder. ECL visualization was performed, and the gel images were analyzed with Image J (NIH image, Bethesda, MD, USA).

### 2.5. Cell Surface Area Measurement

The H9C2 cells were transferred to the laser confocal glass plate, and the density was determined to be moderate. Cells grown to 70–80% confluence were washed twice with PBS and then fixed with 4% paraformaldehyde for 15 min. Afterwards, cells were washed three times with PBS for 5 min, and then 0.1% Triton X-100 was added to the cells at room temperature for 5 min and followed by three additional washes with PBS, each for 5 min. Rhodamine Phalloidin (2.5 *μ*L, R-415 EnzoL; Life Technologies, Waltham, MA, USA) was diluted with PBS to a working solution (200 *μ*L), which was used to stain the cells for 20 min, and then cells were washed three times with PBS for 5 min. Finally, the morphology of the cells was observed under the laser confocal microscope. Ten cells were randomly selected from each group. The length and width of the cells were measured and averaged. The sizes of the cells from each group were compared.

### 2.6. Transmission Electron Microscopy

After the designated treatment, cells for electron microscopic examination were fixed with 2.5% glutaraldehyde and 2.0% paraformaldehyde in 0.1 M sodium cacodylate buffer, overnight at 4°C. The samples were postfixed in 2.0% osmium tetroxide for 1 h at room temperature and then were washed with buffer followed by distilled H_2_O. After being cut with an ultracut microtome, the sections were stained with uranyl acetate and lead citrate. Finally, the section samples were stained with uranyl acetate and lead citrate and examined with an electron microscope fitted with a Hamamatsu digital camera and the AMT Advantage image capture software (Electron Microscopy Resource Lab, University of Pennsylvania, PA, USA).

### 2.7. Cyto-ID® Autophagy Detection Kit

The Cyto-ID Autophagy Detection Kit (0000155258; Promega, Fitchburg, WI, USA) was used to detect the fluorescent molecular probes that have been extensively benchmarked for live cell analysis applications. After 16 h, the cells were divided into 8 groups and then treated with different drugs at different concentrations. After 24 h, the cells in the plate were washed with assay buffer twice. Afterwards, 100 *μ*L of Microscopy Dual Detection Reagent was dispensed so as to cover each sample of the cell monolayer. The samples were protected from light and incubated for 30 minutes at 37°C. Subsequently, the cells were carefully washed with 100 *μ*L of assay buffer, and the excess buffer was removed. The stained cells were analyzed by wide-field fluorescence microscopy. A standard FITC filter set was used for imaging the autophagic signal.

### 2.8. Apoptosis Assay

Cell apoptosis was detected with the Annexin V-FITC Apoptosis Detection Kit (10748800, Hoffmann-La Roche AG, Basel, Switzerland) according to the manufacturer's instructions. After exposure to different experimental conditions for 24 h, cells were harvested, washed twice with cold PBS, and resuspended in binding buffer at a concentration of 1 × 10^6^ cells/mL. Next, the cells were incubated with Annexin V-FITC and PI for 15 min in the dark. Afterwards, samples were analyzed by flow cytometry using the BD FACSCalibur flow cytometer (BD Biosciences, Franklin Lane, NJ, USA). Based on the Annexin V labeling, the apoptotic cells were quantitatively detected at the single-cell level, and the differentiation between apoptotic and necrotic cells was performed.

### 2.9. Cell Viability Assay

Cellular viability was measured using the CellTiter-Glo® Luminescent cell viability assay. Briefly, CellTiter-Glo (Promega) buffer was thawed and equilibrated to room temperature. The lyophilized powder CellTiter-Glo substrate was allowed to equilibrate to room temperature. The CellTiter-Glo Buffer in the appropriate volume (10 mL in pack A, 100 mL in pack B) was transferred into a brown bottle containing the CellTiter-Glo substrate to prepare the enzyme/substrate mixture, which formed the CellTiter-Glo reagents. The H9C2 cardiomyocytes were plated in 96-well plates. After 16 h, the cells were divided into 8 groups and treated with different drugs. After 24 h incubation at 37°C, the plates were incubated at room temperature for 15 minutes. Then, 100 *μ*L of CellTiter-Glo reagent were added to each well and the contents were mixed on an orbital shaker for 2 minutes to induce cell lysis. The 96-well plates were incubated at room temperature for 10 minutes, so that the value of fluorescence signal stabilizes. A microplate reader was used to detect the fluorescence signal.

### 2.10. Statistical Analysis

All experimental data were expressed as the mean ± SD. The data were statistically evaluated using one-way analysis of variance (ANOVA), and a post hoc analysis was performed using the Fisher's least significant difference (LSD) test. The SPSS computer program (version 20.0; IBM Corp., Armonk, NY, USA) was used for the analyses. A *P* value < 0.05 was considered statistically significant.

## 3. Results

### 3.1. Effects of WXKL on AngII-Induced Myocardial Cytoskeletal Protein

**Figure 1:**
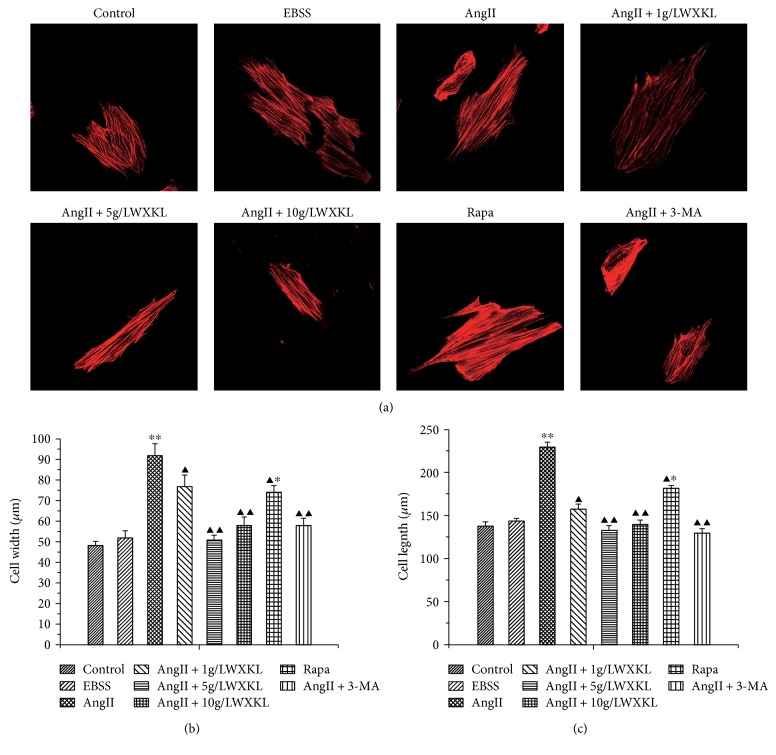
Effect of WXKL on myocardial cytoskeletal proteins induced by AngII. (a) H9C2 cell cytoskeletal proteins. (b) Cell widths was measured by the Image Pro Plus software. (c) Cell lengths of each group of cells measured with the Image Pro Plus software (^∗∗^*P* < 0.01 versus ^∗^*P* < 0.05 versus control group; ^▲▲^*P* < 0.01 versus ^▲^*P* < 0.05 versus AngII).

The cell morphology examination results showed that cell swelling significantly increased after 24 h stimulation with 10^−7^ mol/L. The length and width of the control group cells were clearly increased from 120.56 ± 5.9 *μ*m and 44.57 ± 2.3 *μ*m to 212.43 ± 5.6 *μ*m and 84.36 ± 5.67 *μ*m, respectively, after 24 h stimulation with AngII (*n* = 30, *P* < 0.01; Figures 1(b) and 1(c)). Measurement of the length and width of the cells after treatment with different concentrations of WXKL revealed that WXKL could decrease the AngII-induced length and width from 212.43 ± 5.6 *μ*m and 84.36 ± 5.67 *μ*m to 166.28 ± 5.51 *μ*m and 76.23 ± 5.511 *μ*m (1 g/L), 138.91 ± 6.2 *μ*m and 51.42 ± 2.2 *μ*m (5 g/L), and 145.2 ± 5.3 *μ*m and 57.23 ± 4.1 *μ*m (10 g/L), respectively, (*n* = 30, *P* < 0.01, Figures 1(b) and 1(c)). However, we could also find the same effect with the classical autophagy inhibitor 3-MA, which decreased the length and width of the cells from 212.43 ± 5.6 *μ*m and 84.36 ± 5.67 *μ*m to 125.12 ± 4.8 *μ*m and 59.23 ± 3.5 *μ*m, respectively, (*n* = 30, *P* < 0.05). Compared with the control group, we found that the length and width of the cells induced by Rapa were obviously increased from 120.56 ± 5.9 *μ*m and 44.57 ± 2.3 *μ*m to 178.3 ± 3.41 *μ*m and 71.5 ± 3.421 *μ*m, respectively (*n* = 30, *P* < 0.05, Figures 1(b) and 1(c)).

### 3.2. Effects of WXKL on the Expression of mTOR and LC3 II in Cardiomyocytes Induced by AngII

The expression of mTOR and LC3 II at different concentrations of WXKL was evaluated by Western blot analysis. The results showed that the expression of LC3 II protein was significantly upregulated in the starvation and induced by rapamycin, which was consistent with the effect of 10^−7^ mol/L AngII on LC3 II protein in cardiomyocytes (*P* < 0.05, *n* = 3).

After adding different concentrations of WXKL, we found that it could significantly downregulate the expression of LC3 II protein, which was AngII-induced, and the differences were statistically significant (*P* < 0.01, *n* = 3). However, it did not show a concentration-dependent trend. In addition, we also found that treatment with inhibitors of autophagy, such as 3-MA, could also significantly downregulate the expression of LC3 II protein.

**Figure 2:**
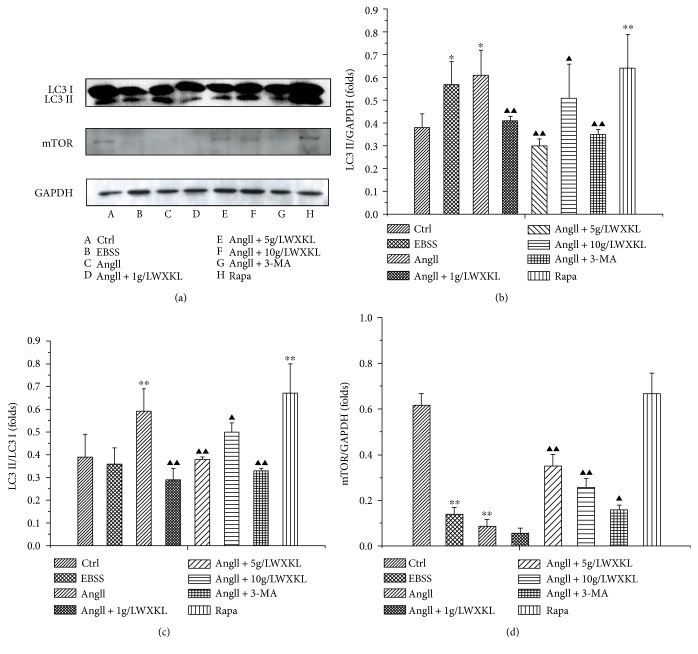
Effect of WXKL on mTOR and MAPLC3A protein expression in AngII-induced cardiomyocytes. (a) The expression levels of mTOR and MAPLC3A protein. (b) The quantification of MAPLC3A in the 8 groups. (c) The quantification of LC3 II/I in the 8 groups. (d) The quantification of mTOR in the 8 groups. Each experiment was repeated three times (^∗^*P* < 0.05, ^∗∗^*P* < 0.01 versus control group; ^▲▲^*P* < 0.01 versus ^▲^*P* < 0.05 versus AngII group).

The same concentration of AngII could downregulate the expression of mTOR protein in the myocardium cells. Treatment with 1 g/L WXKL did not significantly upregulate the expression of mTOR protein compared with 5 g/L and 10 g/L WXKL (*P* < 0.01, *n* = 3). Regarding the expression of mTOR protein, the inhibitory effect was similar to the effect of the classical autophagy inhibitor 3-MA (Figure 2).

### 3.3. Effects of WXKL on the Fluorescence Intensity of LC3 Protein of Autophagy in Cardiomyocytes Induced by AngII

The results showed that compared with the control group, the fluorescence intensity of LC3 protein in cardiomyocytes was significantly increased after AngII treatment, which is consistent with the increase of the fluorescence intensity of LC3 protein after starvation and rapamycin treatment.

**Figure 3:**
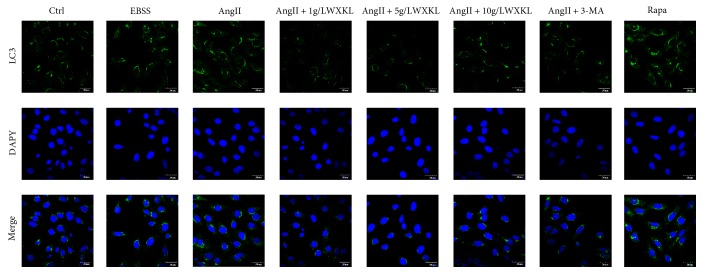
Effect of WXKL on the expression of the autophagy marker protein LC3 in AngII-induced cardiomyocytes. (a) (1–8) shows the staining of LC3 II, (b) (1–8) shows the nucleus, and (c) (1–8) shows the merged images (scale: 30 *μ*m).

Treatment with 3-MA downregulated the AngII-induced fluorescence intensity of LC3, and the different concentrations of WXKL significantly reduced the increase of fluorescence intensity of LC3 induced by AngII, which is consistent with the effect of 3-MA (Figure 3).

### 3.4. Changes of Autophagosomes under Electron Microscope Examination

**Figure 4:**
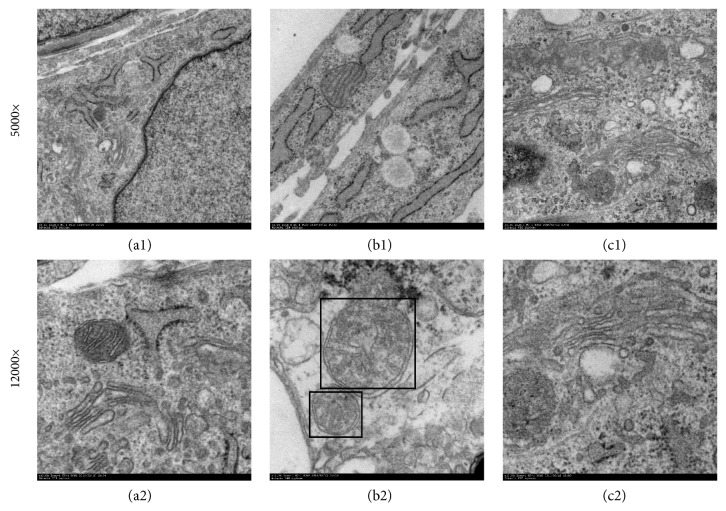
Transmission electron microscopy analysis of cardiomyocytes. (a) The control group (a1: 5000x, a2: 12,000x). (b) The AngII group (a1: 5000x, a2: 12,000x), the black housing showing autophagosomes surrounded by double membranes. (c) The AngII + 5 g/L + WXKL group (a1: 5000x, a2: 12,000x). Scale: 1 *μ*m (a1, b1, and c1), 200 nm (a2, b2, and c2).

Transmission electron microscopy analysis after AngII treatment revealed a large number of aggregated vacuolated structures in the mitochondria and mitochondrial changes, including swelling, cristae rupture, and so forth. Moreover, we could observe numerous autophagosomes. But no autophagosomes were found in the WXKL group. Additionally, the myocardium myofilament and mitochondria were arranged by the WXKL (Figure 4).

### 3.5. Effect of WXKL on the Autophagosomes of AngII-Induced Cardiomyocytes

The fluorescence intensity of autophagosome in the AngII-treated group was significantly higher than that in the control group (*P* < 0.01, *n* = 3), which is consistent with the starvation and rapamycin-induced autophagy groups.

**Figure 5:**
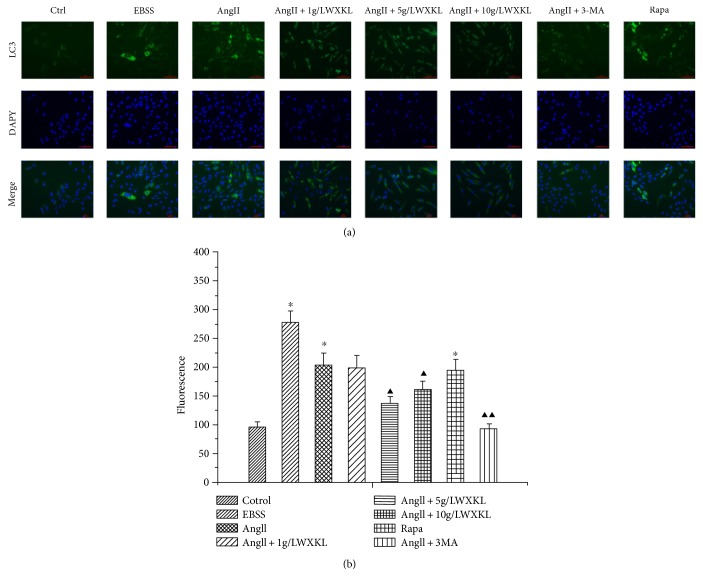
Effect of WXKL on the total amount of autophagosome in AngII-induced cardiomyocytes. (a) Fluorescence after FITC staining. The images of H9c2 cells showing the staining with FITC (A) (1–8), the nucleus (B) (1–8), and the merged images (C) (1–8). (b) The average fluorescence intensity of each group. Each experiment was repeated at least three times (^∗^*P* < 0.05 versus control group; ^▲▲^*P* < 0.01 versus ^▲^*P* < 0.05 versus AngII) (scale: 100 *μ*m).

This effect was also consistent with that of the autophagy inhibitor 3-MA (*P* < 0.01, *n* = 3), and the effect of AngII on autophagy was significantly different (Figure 5).

### 3.6. Effect of WXKL on the Content of ATP

**Figure 6:**
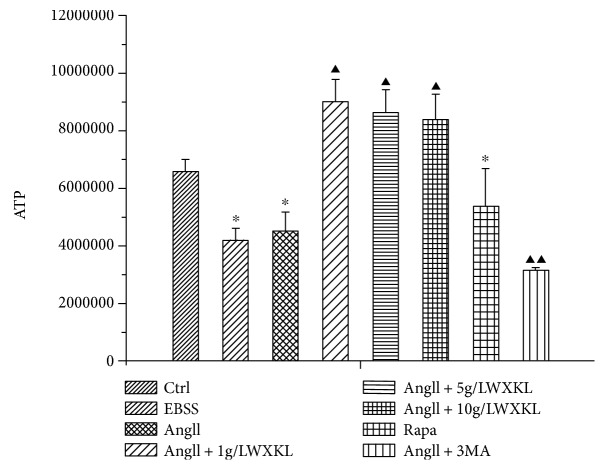
Effect of WXKL on the content of AngII-induced ATP. Cellular viability was measured using the CellTiter-Glo luminescent cell viability assay kit (^∗^*P* < 0.05 versus control group; ^▲^*P* < 0.05; ^▲▲^*P* < 0.01 versus AngII group).

The results showed that the content of ATP in the AngII-treated group decreased by 36% (*n* = 3, *P* < 0.05), compared with the normal cell group, the difference was statistically significant. Comparison of the WXKL-treated groups (1 g/L, 5 g/L, and 10 g/L) with the AngII-treated group revealed that the content of ATP increased by 40%, 36%, and 32%, respectively, (*n* = 3, *P* < 0.01), and these differences were statistically significant (Figure 6).

### 3.7. Effect of WXKL on Apoptosis of Cardiomyocytes Induced by AngII

The results showed that compared with the control group, the apoptotic rate was increased in the AngII group (AngII: 18.14667 ± 3.40148) (*P* < 0.05). Similarly, the apoptotic rate in the starved and rapamycin groups was significantly higher than that in the control group (EBSS: 43.90333 ± 5.41603 versus Ctrl: 8.60667 ± 2.24915, Rapa: 21.35333 ± 5.5634 versus Ctrl: 60667 ± 2.24915, *n* = 3, *p* < 0.05), and the differences were statistically significant.

**Figure 7:**
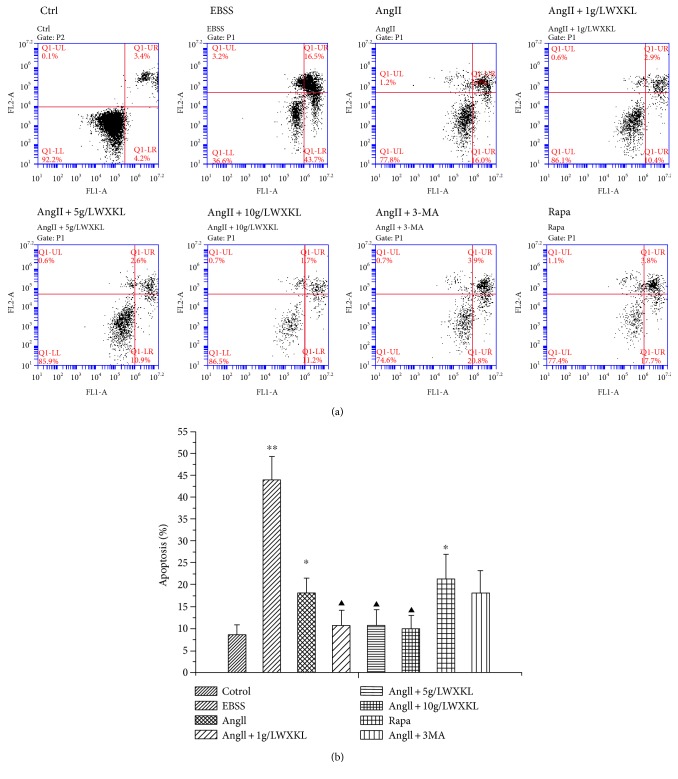
Effect of WXKL on AngII-induced cardiomyocyte apoptosis. (a) Flow cytometry-based apoptosis assay for H9C2 cell. (b) The statistical quantification of cell apoptosis, triplicate experiments were conducted for each group (^∗∗^*P* < 0.01; ^∗^*P* < 0.05 versus control group; ^▲c^*P* < 0.01 versus AngII group).

The addition of WXKL inhibited the apoptosis induced by AngII, and the difference was statistically significant (*n* = 3, *p* < 0.05). On the other hand, the autophagy inhibitor 3-MA had no effect on the AngII-induced apoptosis (Figure 7).

## 4. Discussion

The main findings of the current study are (1) AngII induces autophagy through upregulating the expression of LC3 II protein, decreasing the intracellular ATP content, and promoting the apoptosis of cardiomyocytes. Additionally, AngII-induced autophagy may be dependent on the activation of mTOR. (2) WXKL inhibits AngII-induced autophagy, the mechanism may be by reducing the expression of LC3 II protein, increasing the production of ATP, and reducing the apoptosis of myocardial cell. WXKL-mediated cell survival is, in part, mediated through the inhibition of autophagy, involving the mTOR pathway.

Cardiac hypertrophy is a common pathological change that increases the incidence and mortality of many cardiovascular diseases. Although there are many factors and regulators that affect myocardial hypertrophy, the renin-angiotensin (RAS) and its primary effector peptide, AngII, are involved in the pathophysiology of cardiac hypertrophy and failure.

Recently, several studies have shown that autophagy plays important roles in AngII-induced cardiac hypertrophy. Some researchers have shown that AngII treatment induces autophagy, whereas others have shown that AngII treatment inhibits autophagy.

Our results show that AngII could result in autophagy of the H9C2 cells. Meanwhile, WXKL inhibited AngII-induced autophagy. In this study, we examined the expression of the LC3 II protein by confocal laser scanning confocal microscope. We found that the fluorescence intensity of the LC3 II protein was increased in AngII-induced cardiomyocytes and abundant in intracellular and pericentric regions. In addition, quantitative analysis of autophagy, with the Cyto-ID autophagy test kit, showed that AngII increases autophagy fluorescence intensity. As a marker of autophagic activity, growing evidence highlights the importance of LC3B-II/I protein in monitoring autophagy in myocardial hypertrophy. Previous research showed that AngII (1 *μ*mol/L for 48 h) induced neonatal rat cardiomyocytes hypertrophy, accompanied by a marked increase in cell area and expression of ANP and *β*-MHC. In addition, the expression of the LC3 II/I protein and mRNA was also upregulated. Moreover, overexpression of the LC3 II/I protein resulted in upregulated levels of autophagic activity and increased ANP and *β*-MHC mRNA expression as well as increased cardiomyocyte area in the AngII treatment cardiomyocytes. Our results indicate that AngII upregulates the expression of LC3B-II/I protein, accompanied by a marked increase in cell area. Accordingly, we hold that LC3 II/I-mediated autophagy plays a role in the regulation of myocardial hypertrophy induced by AngII, which may provide a therapeutic target to reverse myocardial hypertrophy induced by AngII. Compared to the AngII group, WXKL inhibited the AngII-induced autophagic response in the heart. Thus, WXKL could be a negative regulator of pathological autophagy and cardiac remodeling.

This study found that AngII reduced the production of ATP, while WXKL counteracted the inhibition of ATP production by AngII. Under normal circumstances, the heart can use a variety of energy substrates, they play a crucial role in the energy metabolism of the heart, but when myocardial hypertrophy occurs, the energy substrate will appear decompensated. Usually when the heart needs energy, creatine phosphate (CP), an energy storage phosphagen, and adenosine diphosphate (ADP) will quickly be converted to adenosine triphosphate (ATP) and creatine, thus CP/ATP is commonly used as a measure of energy balance standards. Studies have shown that ATP deficiency leads to an increase in cell autophagy, which is consistent with our findings.

The regulatory process underlying autophagy is very complex and involves many signaling molecules. mTOR associates with other proteins and functions as a core component of two different protein complexes, mTOR complex 1 and mTOR complex 2, which regulate distinct cellular processes. Specifically, as a core component of both complexes, mTOR serves as a serine/threonine protein kinase that controls cell growth, cell proliferation, cell motility, cell survival, protein synthesis, autophagy, and transcription. As a core component of mTORC2, mTOR also serves as a tyrosine protein kinase that promotes the activation of insulin receptors and insulin-like growth factor 1 receptors. mTORC2 has also been connected with the regulation and maintenance of the actin cytoskeleton. Through these various activities, mTOR integrates the input from upstream pathways, including insulin, growth factors (such as IGF-1 and IGF-2), and amino acids. In this way, mTOR senses cellular nutrient, oxygen, and energy levels. Accordingly, the activity of mTOR is regulated by intracellular nutritional status (amino acid), growth factor (insulin), and cellular energy state (ATP). In particular, mTOR is a pivotal upstream mediator of autophagy through its binding and inactivation of the autophagy kinase complex ULK1/2, thereby blocking the formation of autophagosomes [17]. mTOR has also been found to have an important role in the maintenance of the cardiac function [18]. The cardiomyocyte-specific deletion of mTOR results in severe dilated cardiomyopathy, whereas the pharmacological inhibition of mTOR reverses cardiac hypertrophy [19, 20]. Our results show that WXKL can significantly prevent the AngII-induced downregulation of the mTOR protein in vitro. Thus, our study demonstrated that WXKL could reverse heart failure by regulating mTOR activity and autophagy and provided further evidence that an appropriate level of autophagy is essential for the maintenance of cardiac function.

Several mechanisms may contribute to the WXKL-mediated inhibition of pathological autophagy. Autophagy and apoptosis are two physiological processes that take place inside the cell to eliminate old or damaged cells for recovery of the effective protein component. On the one hand, autophagy, as a mechanism of cell survival, can remove toxic substances and damaged proteins, organelles (such as mitochondria, ribosomes, and endoplasmic reticulum), and in the case of undernutrition, hypoxia and recycling degradation, these substances release amino acids, nucleotides, fatty acids, and other small molecules and energy to maintain the stability of the intracellular environment. In some cases, this process can also lead to cell death, known as type II programmed cell death. On the other hand, apoptosis can remove damaged and unwanted cells. Apoptosis is caspase-dependent and involves cell shrinkage, chromatin condensation, apoptotic bodies, and so forth [21]. In some experiments with cardiomyocytes or cardiomyocyte hypoxia/reoxygenation models, the induction of autophagy is thought to have an apoptotic effect [22]. However, other studies have suggested that autophagy has a protective effect and upregulation of autophagy can attenuate myocardial ischemia and reperfusion (MIRI) [23]. In our study, we found that treatment with rapamycin, a well-established inducer of autophagy, enhanced the autophagosome formation and induced cardiomyocyte apoptosis. Further, treatment with 3-MA, which inhibits the initial steps of the autophagic process, attenuated the autophagic puncta and autophagosome formation induced by WXKL treatment. These results suggest that AngII can induce cardiomyocyte apoptosis and autophagy, while the inhibitor 3-MA and WXKL can inhibit the AngII-induced myocardial cell apoptosis. But its mechanism needs further study.

Autophagy salvages the dysfunctional protein and organelles. Defects in this process can lead to accumulation of proteins and organelles that can be toxic for cells and lead to autophagic cell death. Excessive autophagy in the heart can cause nonselective degradation of proteins and organelles and thus functional defects in cardiac cells and ultimately heart failure [24]. Therefore, strict regulation of autophagy is critical during disease condition. Zhu et al. reported [25] that beclin-1 overexpression exaggerated autophagic activity and accentuated pathological remodeling. Heterozygous disruption of this gene decreased cardiomyocyte autophagy and diminished load-induced pathological remodeling. Weng et al. [26] performed a transverse aortic constriction (TAC) surgery in mice and found that TAC caused marked heart hypertrophy and promoted cardiac autophagy in the heart. Autophagic structures, Atg5, and Atg16 mRNA expression levels as well as LC3 II and beclin-1 protein levels are all increased in the TAC-treated group through the PKC and ERK1/2 pathways. Here, several standard experiments for detecting autophagy, including transmission electron microscopy, confocal microscopy, and Western blot analysis, were conducted to demonstrate that WXKL treatment significantly inhibited the deleterious effects of AngII and attenuated autophagy in vitro.

In conclusion, to our knowledge, this is the first study describing a novel role of WXKL in pathological autophagy. In sum, our study found that AngII can enhance the activity of mTOR, upregulate the content of LC3 II protein, induce autophagy in cardiomyocytes, decrease the intracellular ATP content, and promote the apoptosis of cardiomyocytes induced by autophagy. WXKL can inhibit the activity of mTOR, reduce the expression of LC3 II protein, and inhibit AngII-induced autophagy of cardiomyocytes thereby increasing the generation of ATP and inhibition of myocardial cell apoptosis. We propose that a novel role of WXKL is the regulation of pathological autophagy. Thus, WXKL can act as a potential therapeutic molecule for the treatment of chronic heart disease.
